# Supplementary material for: Ribosome Profiling and RNA Sequencing Reveal Translation and Transcription Regulation under Acute Heat Stress in Rainbow Trout (Oncorhynchus mykiss, Walbaum, 1792) Liver
Source: Int J Mol Sci. 2024 Aug 14;25(16):8848. doi: 10.3390/ijms25168848 (PMC11354268; doi:10.3390/ijms25168848)
Supplement: Supplementary file 1 [file ijms-25-08848-s001.zip › supplementary figures and Tables legends.pdf]

SI legends

Figure S1. Pearson correlation coefficients between the gene translational level and transcriptional level under heat stress.

Figure S2. GO annotation of discordantly regulated genes under heat stress condition. (A) GO annotation of genes down-regulated at transcriptional level and unchanged at translational level (Class D). (B) GO annotation of genes up-regulated at transcriptional level and unchanged at translational level (Class F). (C) GO annotation of genes down-regulated at translational level and unchanged at transcriptional level (Class H). (D) GO annotation of genes up-regulated at translational level and unchanged at transcriptional level (Class B). P-value of GO terms was under the control of FDR < 0.05.

Figure S3. The impact of sequence characteristics on four TE groups in the (A) 3'UTR. (B) 5'UTR, and (C) CDS regions of HS samples. The letters a-d highlight groups with significant differences, as determined by the x Student's t-test with $p < 0.05$.

Figure S4. Translated uORFs affects the translation of mORFs. (A) comparison of uORFs lengths, (B) comparison of 5'UTR length, (C) comparison of uORFs NMFE, (D) Normalized distance to CDS start, (E) Normalized distance to the transcription start site (TSS) between translated and untranslated sequences in the HS samples. *p*-values were tested by Student's t test.

Table S1. Alignment statistics for Ribosome profiling reads.

Table S2. Alignment statistics for RNA-seq reads.

Table S3. List of genes in four main discordant responsive groups.

Table S4. Numbers of gene analyzed in 4 different TE (translational efficiency) groups.
