# Supplementary figures and images for: Ribosome Profiling and RNA Sequencing Reveal Translation and Transcription Regulation under Acute Heat Stress in Rainbow Trout (Oncorhynchus mykiss, Walbaum, 1792) Liver
Source: Int J Mol Sci. 2024 Aug 14;25(16):8848. doi: 10.3390/ijms25168848 (PMC11354268; doi:10.3390/ijms25168848)

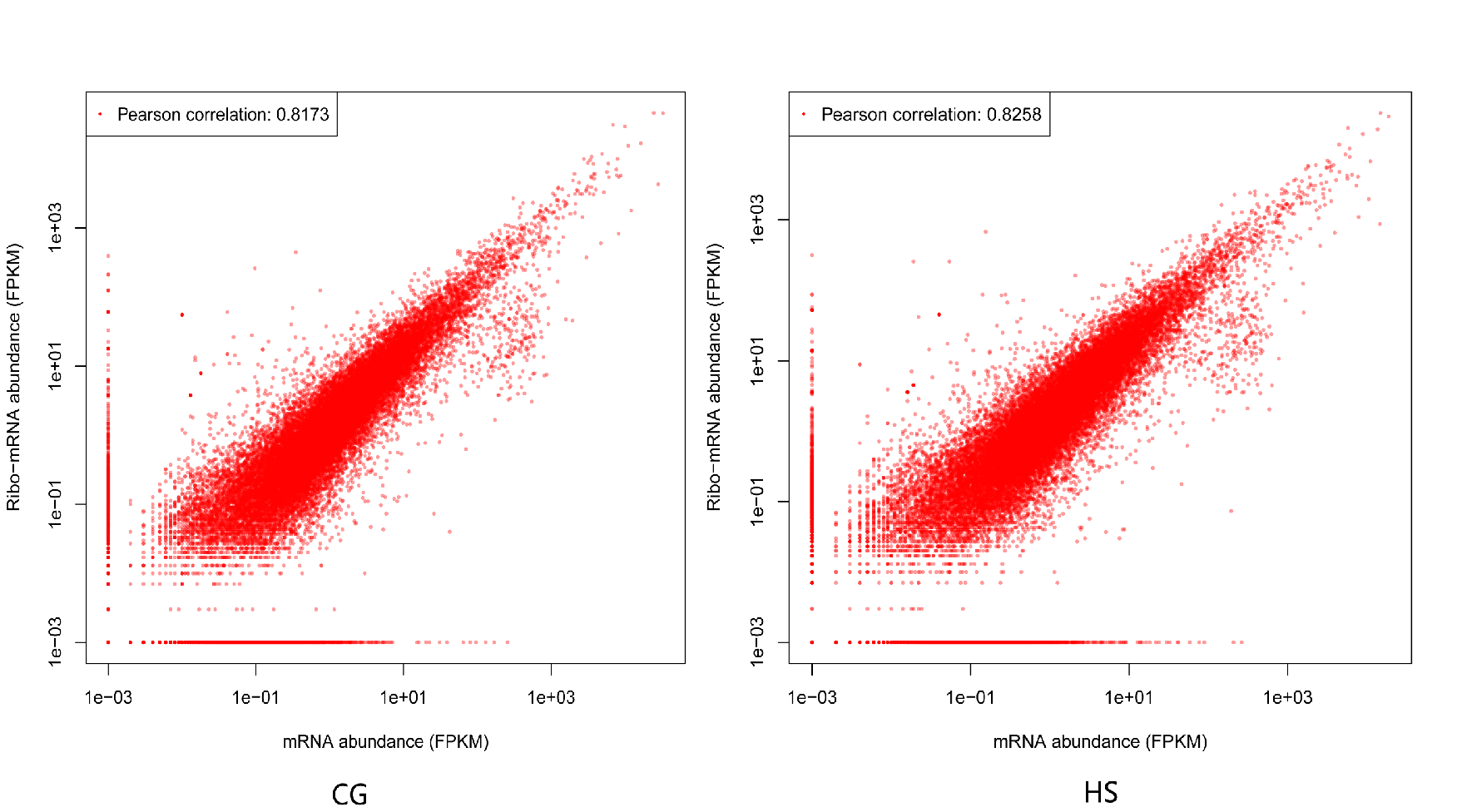

Supplement: Supplementary file 1 [file ijms-25-08848-s001.zip › Figure S1.tif]

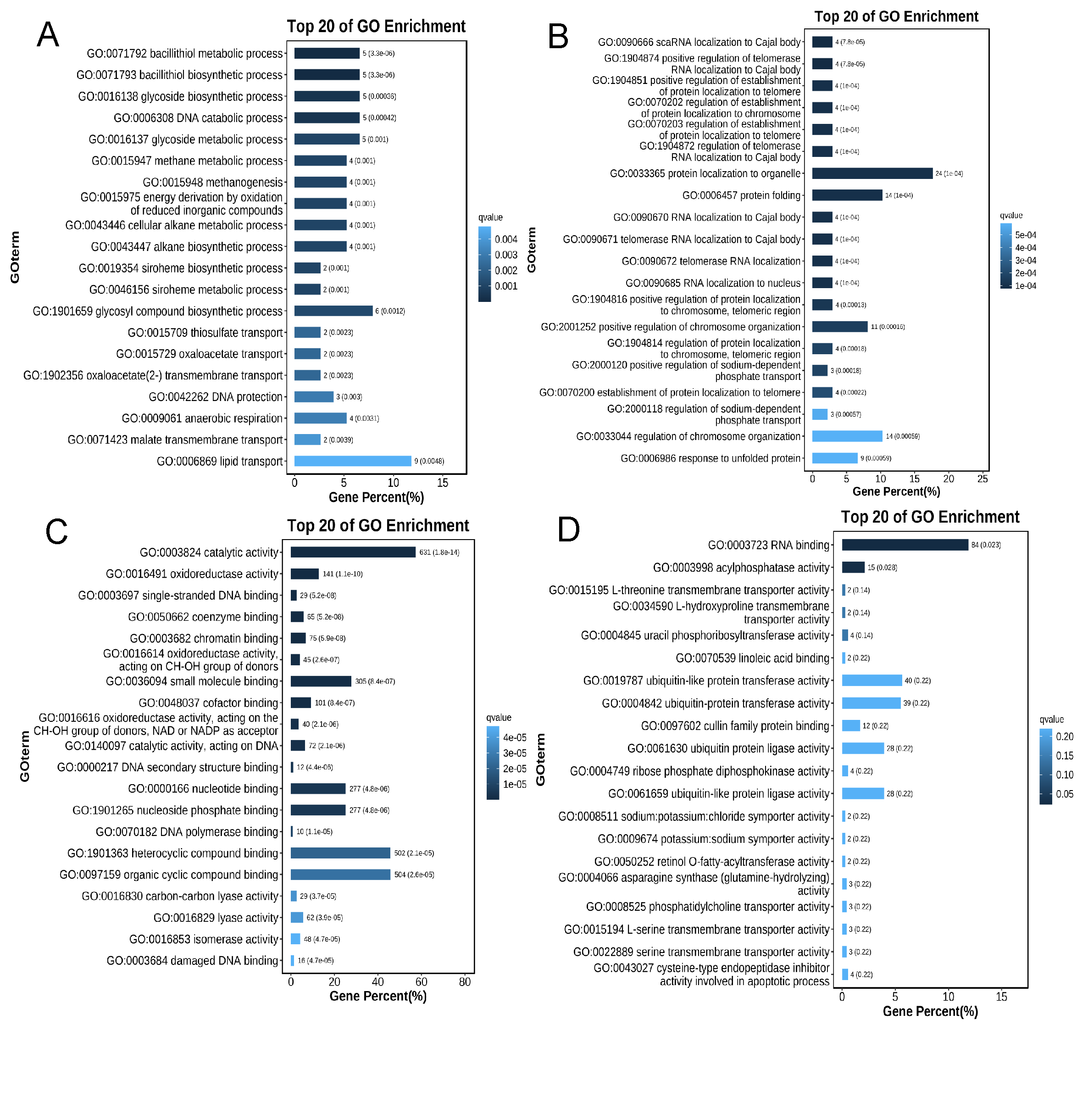

Supplement: Supplementary file 1 [file ijms-25-08848-s001.zip › Figure S2.tif]

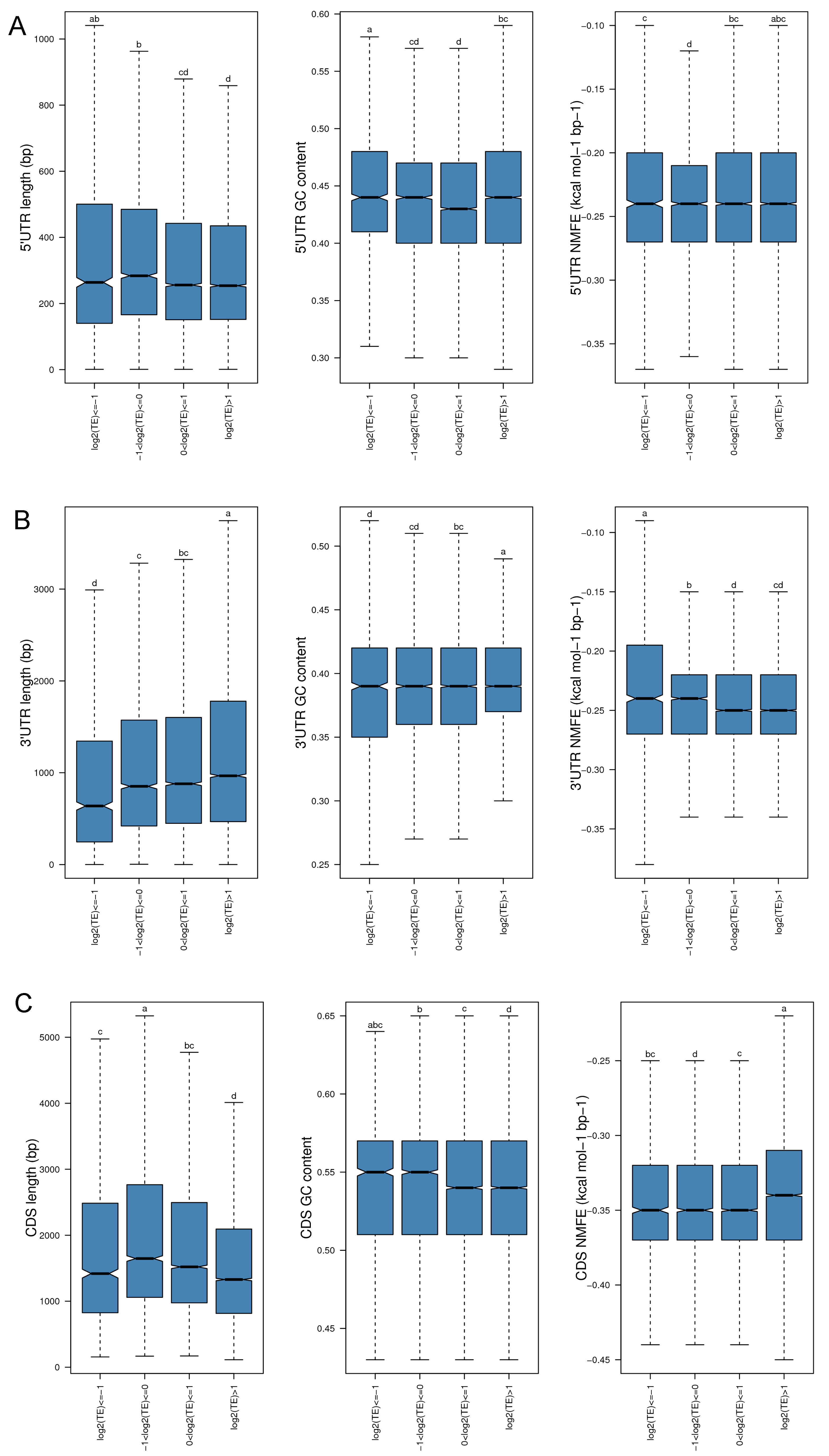

Supplement: Supplementary file 1 [file ijms-25-08848-s001.zip › Figure S3.tif]

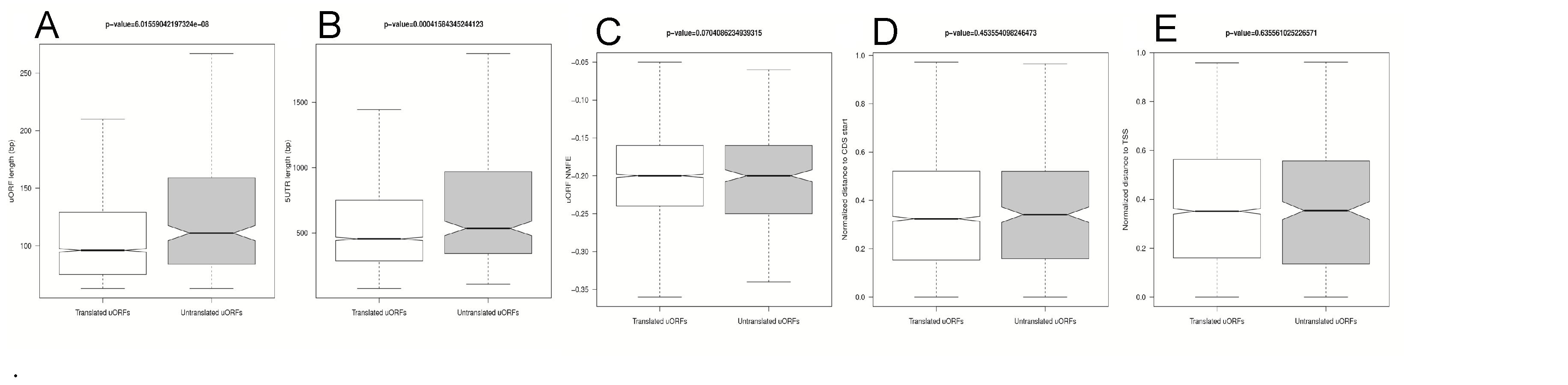

Supplement: Supplementary file 1 [file ijms-25-08848-s001.zip › Figure S4.tif]
